# Physiological, mitochondrial, and oxidative stress differences in the presence or absence of lactation in rats

**DOI:** 10.1186/s12958-017-0317-7

**Published:** 2018-01-09

**Authors:** Hayden W. Hyatt, Yufeng Zhang, Wendy R. Hood, Andreas N. Kavazis

**Affiliations:** 1 School of Kinesiology, Auburn University, 301 Wire Road, Auburn, Alabama 36849 USA; 2 Department of Biological Sciences, Auburn University, Auburn, AL USA

**Keywords:** Lactation, Metabolism, Mitochondria, Oxidative stress

## Abstract

**Background:**

Human epidemiological data show that breastfeeding reduces the mother’s probability of developing several disease conditions, including obesity and type II diabetes compared to mothers that give birth but do not breastfeed. The goal of this investigation was to characterize how lactation changes a rat’s body composition, metabolism, mitochondrial function, and oxidative stress.

**Methods:**

Ten-week old female Sprague-Dawley rats were divided into three groups (*n* = 8 per group): 1) non-reproductive (NR), 2) those that were allowed to mate and give birth, but were not allowed to suckle their pups (PP), and 3) those that were allowed to mate and give birth, and suckled their young until weaning at 21 days (PL). All animals were sacrificed at a time corresponding to 7 days following the weaning of pups (i.e., day 28 postpartum).

**Results:**

The body mass of PL rats was similar to NR rats, but the body mass of PP rats was higher than NR rats. Importantly, PL rats had lower retroperitoneal white adipose tissue mass compared to both NR and PP rats. The difference in fat mass was accompanied by higher protein levels of PPARδ, SOD2, and reduced oxidative damage. Furthermore, the liver of PL rats had higher mitochondrial function with NADH-linked substrates, and higher expression of PGC-1α, PPARδ, and SOD2.

**Conclusions:**

These acute differences observed between female rats that did and did not suckle their young could be used as the foundation for future research investigating the prolonged and sustained benefits of lactation.

## Background

Exercise, fasting, and calorie restriction are examples of metabolic perturbations that induce physiological adaptations capable of promoting a healthy phenotype that is protective against various health disparities [1–4]. However, investigators often overlook lactation as a challenging metabolic event. Indeed, epidemiological studies in humans suggest that breastfeeding benefits the mother by reducing the risk of obesity, type II diabetes, hypertension, and several types of cancer [5–9]. Yet, why these health benefits may accrue to breastfeeding are largely unknown.

During reproduction, a female’s metabolism goes through two dramatic changes. With the onset of pregnancy, a female’s metabolic processes are primed to support glucose and amino acid transport to the fetus. Glucose transport is facilitated by an increase in maternal insulin production and as a result, maternal cells often display increased resistance to insulin [10, 11]. This change could also be associated with increases in circulating fatty acids and increased visceral adiposity [12]. In contrast, during lactation there is a decrease in insulin secretion, which is associated with a drop in β-cell proliferation, improved insulin sensitivity, and a shift in lipoprotein lipase and triacylglyceride levels that facilitate the mobilization of lipid precursors to be used for milk synthesis [13, 14]. Based on current epidemiological data in humans and the limited physiological observations in animal models, Stuebe and Rich-Edwards proposed the reset hypothesis which posits that lactation plays a central role in mobilizing fat stores and resetting the risk of metabolic disease [9]. However, research evaluating the physiological differences between females that give birth and lactate versus those that give birth and do not participate in lactation are lacking.

White adipose tissue (WAT), liver, and skeletal muscle account for more than 50% of a non-reproductive adult’s metabolic rate [15]. Thus, any physiological changes that regulate metabolism in these organs during lactation could contribute to potential protective effects conferred by lactation. In this regard, Gutgesell and collaborators showed that genes important in oxidative and lipid metabolism (i.e. peroxisome proliferator activated receptor alpha [PPAR-α], peroxisome proliferator activated receptor gamma coactivator 1 alpha [PGC-1α], and peroxisome proliferator activated receptor gamma coactivator 1 beta [PGC-1β]) displayed reduced mRNA expression in liver and skeletal muscle 2 weeks following the cessation of lactation when compared to animals that gave birth but did not suckle their young [16]. Additionally, Pichaud and collaborators reported decreased electron transport system activity in the liver during peak lactation in the mouse [17]. Mediation of oxidative metabolism and thus mitochondrial function also may have important health implications on states of oxidative stress. As such, the current study seeks to delineate the potential effects that lactation has on markers of metabolism, mitochondrial function, and oxidative stress. Specifically, we hypothesized that females that give birth and suckle their pups would have lower body mass due to enhanced lipid metabolism and lower blood glucose concentrations driven, in part, by enhanced mitochondrial signaling in liver and skeletal muscle. In addition, we hypothesized that the higher metabolic demand in lactation would not impose an increase in markers of oxidative damage, as suggested to be a consequence of reproduction [18].

## Methods

### Animal husbandry

All experimental procedures were approved by Auburn University’s Institutional Animal Care and Use Committee (PRN 2014–2591). Ten-week old Sprague-Dawley rats were obtained from Envigo (Indianapolis, IN). Animals were acclimated with their diet and facility 10 days prior to the beginning of the experiment. Rats were housed under standard laboratory conditions (46 × 25 × 20 cm boxes, 12 L:12D cycle, 22°C, 50% RH), and given ad libitum access to food (Teklad Global Diet 2018, Envigo) and water. Female rats (*n* = 8 per group) were randomly assigned to one of three treatment groups: 1) non-reproductive (NR), 2) those that were allowed to mate and became pregnant, but were not allowed to suckle their pups (PP), and 3) those that were allowed to mate, became pregnant, and suckled their young for 21 days (PL). Female rats were paired in their boxes with same-group counterpart, but separated during late pregnancy and lactation to prevent cross-fostering of pups after birth. Pups were removed from females in the PP group within 12 h of birth. Litter size was adjusted to eight on the day of parturition pups, and were weaned and removed from the box on day 21 for females in the PL group. All animals were sacrificed at a time corresponding to 7 days following the weaning of pups (i.e., day 28 postpartum), allowing PL females to return to a non-reproductive state.

### Blood collection and analysis

Rats were fasted for 4 h and then were anesthetized using isoflurane vapors and body mass was quickly recorded. The anesthetized animals were than decapitated, and blood was collected, allowed to clot, and then centrifuged. Following centrifugation the serum was frozen at −80 °C for subsequent analyses. Serum glucose (STA-680, Cell Biolabs, San Diego, CA, USA) and non-esterified fatty acids (NEFA) (STA-618, Cell Biolabs) were quantified using the manufacturer’s specifications.

### Tissue collection and handling

After the decapitation, the following tissues were excised and weighed: liver, triceps surae (calf muscle), and the retroperitoneal white adipose tissue (WAT). After the mass of each tissue was recorded, a sample of tissue from calf skeletal muscle and liver was used for mitochondrial isolation and the remainder of tissues were frozen in liquid nitrogen and stored at −80 °C for subsequent analyses. Upon gross evaluation, mammary tissue was not observed for females in any of the groups suggesting that the regression of mammary tissue was nearly or fully complete for females in the PP and PL groups at the time of tissue collection.

### Mitochondrial isolation

Mitochondrial isolations for muscle were performed as previously described [19]. Excised muscles (~750 mg) were trimmed to remove fat and connective tissues, weighed, and placed in 10 volumes of solution I (100 mM KCl, 40 mM Tris HCl, 10 mM Tris base, 1 mM MgSO4, 0.1 mM EDTA, 0.2 mM ATP, and 2% (wt/vol) free fatty acid bovine serum albumin (BSA), pH 7.40). Muscles were minced with scissors and the mince was homogenized for 15 s with a polytron. Protease (Trypsin) was added (5 mg/g wet muscle), and the digested mince was mixed continually for 7 min. Digestion was terminated by the addition of an equal volume of solution I. The homogenate was centrifuged at 500 *g* for 10 min at 4 °C and the supernatant was rapidly decanted through a double layer of cheesecloth and centrifuged at 3500 *g* for 10 min at 4 °C. The supernatant was discarded and the mitochondrial pellet was resuspended in solution I. The suspension was centrifuged at 3500 *g* for 10 min at 4 °C. The supernatant was again discarded, and the pellet was resuspended in 10 volumes of solution II (similar to solution I, but without BSA). This resuspended pellet was subsequently centrifuged at 3500 *g* for 10 min at 4 °C. The final pellet containing mitochondria was suspended in 250 μl of a solution containing 220 mM mannitol, 70 mM sucrose, 10 mM Tris HCl, and 1 mM EGTA, pH 7.40. Mitochondria from liver were isolated as previously described [20]. Briefly, liver (~750 mg) was weighed and placed in 10 volumes of solution III (250 mM sucrose, 5 mM HEPES, and 1 mM EGTA), minced with scissors and the mince was homogenized with a Potter-Elvehjem PTFE pestle and glass tube (2 passes). The homogenate was centrifuged at 500 g for 10 min at 4 °C and the supernatant was rapidly decanted through a double layer of cheesecloth and centrifuged at 3500 g for 10 min at 4 °C. The supernatant was discarded and the mitochondrial pellet was resuspended in solution III. The suspension was centrifuged at 3500 g for 10 min at 4 °C. The final pellet containing mitochondria was suspended in 250 μl of a solution containing (in mM) 220 mannitol, 70 sucrose, 10 Tris HCl, and 1 EGTA, pH 7.40. All procedures we performed on ice and all solutions were kept on ice (4 °C).

### Isolated mitochondrial oxidative phosphorylation

Mitochondrial oxygen consumption was measured as described by Messer et al. [21]. Briefly, mitochondrial oxygen consumption was measured polarographically in a respiration chamber (Hansatech Instruments, United Kingdom). Isolated mitochondria (20 μL) were incubated with 1 ml of respiration buffer adapted from Wanders et al. [22] (100 mM KCL, 50 mM MOPS, 10 mM KH_2_PO_4_, 20 mM glucose, 10 mM MgCl_2_, 1 mM EGTA, and 0.2% fatty acid free BSA; pH = 7.0) at 37 °C in a respiratory chamber with continuous stirring. For state 3 respiration, 2 mM pyruvate and 2 mM malate (complex I substrates) or 5 mM succinate (complex II substrate) was used in the presence of 0.25 mM ADP, and state 4 respiration was recorded following the phosphorylation of ADP as described by Estabrook [23]. Respiratory control ratio (RCR) was calculated as state 3/state 4 oxygen consumption. Respiration values were expressed as a ratio to citrate synthase to compensate for mitochondrial enrichment in the samples.

### Mitochondrial oxidant emission

Oxidant emission by mitochondria was determined using the oxidation of the fluorogenic indicator Amplex Red (Molecular Probes, Eugene, OR) in the presence of horseradish peroxidase [24]. The assay was performed at 37 °C in 96-well plates using succinate as the substrate. Specifically, this assay was developed based on the concept that horseradish peroxidase catalyzes the hydrogen peroxide-dependent oxidation of nonfluorescent Amplex Red to fluorescent Resorufin Red. Resorufin Red formation was monitored at an excitation wavelength of 545 nm and an emission wavelength of 590 nm using a multiwell plate reader fluorometer (Synergy H1, BioTek, Winooski, VT, USA). We recorded the level of Resorufin Red formation, and hydrogen peroxide production was calculated with a standard curve.

### Enzymatic assays for electron transport chain complex activity

Complex I (NADH dehydrogenase) enzyme activity (EC 1.6.5.3) was measured as a function of the decrease in absorbance from NADH oxidation by decylubiquinone before and after rotenone addition [25]. Complex II (succinate dehydrogenase) activity (EC 1.3.5.1) was measured as a function of the decrease in absorbance from 2,6-dichloroindophenol reduction [25]. Complex III (ubiquinol cytochrome *c* oxidoreductase) activity (EC 1.10.2.2) was determined as a function of the increase in absorbance from cytochrome *c* reduction [25]. Complex IV (cytochrome *c* oxidoreductase) activity was determined as a function of the decrease in absorbance from cytochrome *c* oxidation [25]. Specificity of complex IV activity was determined by monitoring changes in absorbance in the presence of KCN [25]. Citrate synthase (EC 4.1.3.7) was measured as a function of the increase in absorbance from 5,5′-dithiobis-2-nitrobenzoic acid reduction [25]. Enzyme activities were expressed as a ratio to citrate synthase to compensate for mitochondrial enrichment in the cell samples. All measurements were performed using the BioTek Synergy H1 spectrophotometer (BioTek, Winooski, VT, USA).

### Protein abundance

The relative concentration of proteins was quantified by Western blot analysis [24]. To accomplish this, tissue was homogenized 1:10 (wt/vol) in 5 mM Tris HCl (pH 7.5) and 5 mM EDTA (pH 8.0), and protease inhibitor cocktail (14224–396, VWR, Radnor, PA, USA) and was centrifuged at 1500 *g* for 10 min at 4 °C. Protein content of the supernatant was quantified by the method of Bradford [26]. Proteins were separated by polyacrylamide gel electrophoresis via 4–20% polyacrylamide gels (BioRad, Hercules, CA, USA). After electrophoresis, the proteins were transferred to PVDF membranes. Non-specific sites were blocked in phosphate-buffered saline (PBS) solution containing 0.1% Tween 20 and 5% non-fat milk. Membranes were then incubated overnight at 4 °C with primary antibodies purchased from GeneTex (Irvine, CA, USA) directed against peroxisome proliferator activated receptor alpha (PPARα, GTX101096, 1:1000), peroxisome proliferator activated receptor delta (PPARδ, GTX113250, 1:2000), peroxisome proliferator activated receptor gamma, coactivator 1 alpha (PGC-1α, GTX37356, 1:1000), superoxide dismutase 1 (SOD1, GTX100554 1:2000), superoxide dismutase 2 (SOD2, GTX116093, 1:2000), catalase (CAT, GTX110704, 1:2000), and glutathione peroxidase (GPX, GTX116040, 1:2000). Following incubation with primary antibodies, membranes were washed with PBS-Tween (5 min × 3) and then incubated with secondary antibodies for 1 h in room temperature. After washing (5 min × 3), a chemiluminescent system was used to detect labeled proteins (GE Healthcare, Buckinghamshire, UK). Images of the membranes were captured and analyzed by using the ChemiDoc-It2 Imaging System (UVP, LLC, Upland, CA). Protein expression was normalized to Ponceau staining.

### Assessment of indices of oxidative damage

To determine the relative amount oxidative damage, we measured protein oxidation and lipid peroxidation. Lipid peroxidation was assessed by determining 4-hydroxynoneal (4-HNE; *trans*-4-hydroxy-2-nonenal, C_9_H_16_O_2_) expression via western blotting, as described above. Primary antibody for 4-HNE was purchased from Abcam (ab46545; 1:1000 dilution, Cambridge, MA, USA). Protein oxidation was measured by comparing relative expression of protein carbonyls using a commercially available kit (Oxy-Blot protein oxidation detection kit; Intergen, Purchase, NY, USA) via western blotting, as described by the manufacturer’s instructions.

### Statistics

Comparison between groups for each dependent variable were made by a one-way analysis of variance (ANOVA), with a Tukey post hoc test being used to determine significance differences between groups. However, in the case of state 3 respiration with succinate in muscle and serum FFA, the Brown-Forsythe test was significant and thus the Kruskal-Wallis test was performed, followed by the Dunn’s post-hoc to determine significance differences between groups. Data are presented as mean ± SD, and significance was established at *p* < 0.05.

## Results

### Body and tissue mass

**Fig. 1:**
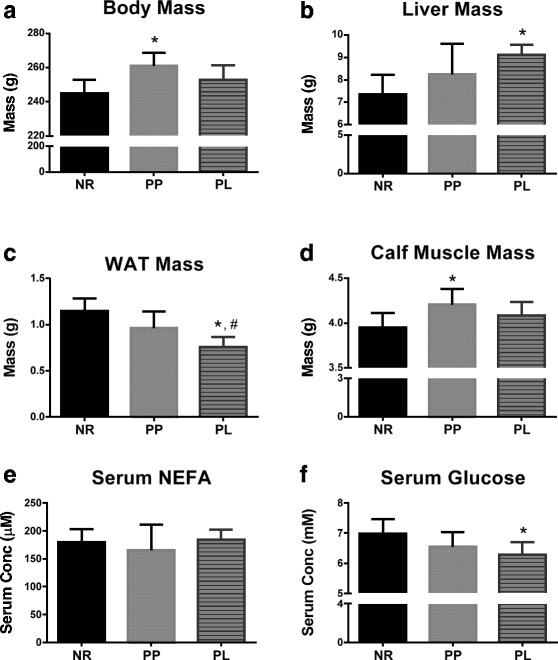
Body mass, tissue mass, and serum metabolites for age-matched rats that did not reproduce (NR), and rats that did not (PP) or did (PL) suckle their young for 21 days postpartum. **a** Body mass, (**b**) liver mass, (**c**) retroperitoneal white adipose tissue (WAT) mass, (**d**) mass of both rear triceps surae (calf muscle mass), (**e**) serum concentration of non-esterified fatty acids (NEFA), and (**f**) serum concentration of glucose. Data shown are mean ± SD. * indicates different from NR (*p* < 0.05), and # indicates different from PP (*p* < 0.05)

During pregnancy and lactation, the body of females undergoes a large fluctuation in mass to accommodate the developing fetuses and active mammary tissue. The regression of these tissues 28 days following pregnancy and lactation varied between groups. Body mass was higher in PP compared to NR rats (*p* = 0.005) and the body mass of PP rats was higher than the mass of the PL rats, but this was not statistically different (*p* = 0.145) (Fig. 1a). Liver mass was higher in PL than NR rats (*p* = 0.004) (Fig. 1b). PL rats had lower retroperitoneal fat mass compared to both PP (*p* = 0.041) and NR rats (*p* < 0.001) (Fig. 1c). The combined mass of the rear triceps surae calf muscle was higher in PP than in NR rats (*p* = 0.027) (Fig. 1d).

### Serum glucose and NEFA concentrations

The high energetic demand of lactation requires energy substrates to be delivered to metabolically active tissues via the blood stream. Two important metabolites transported in blood are glucose and NEFAs. Serum glucose concentration was lower in PL compared to NR (*p* = 0.029), but no statistical differences were detected in serum NEFA concentrations (*p* = 0.802) (Fig. 1e and f).

### Mitochondrial respiration and oxidant emission

**Fig. 2:**
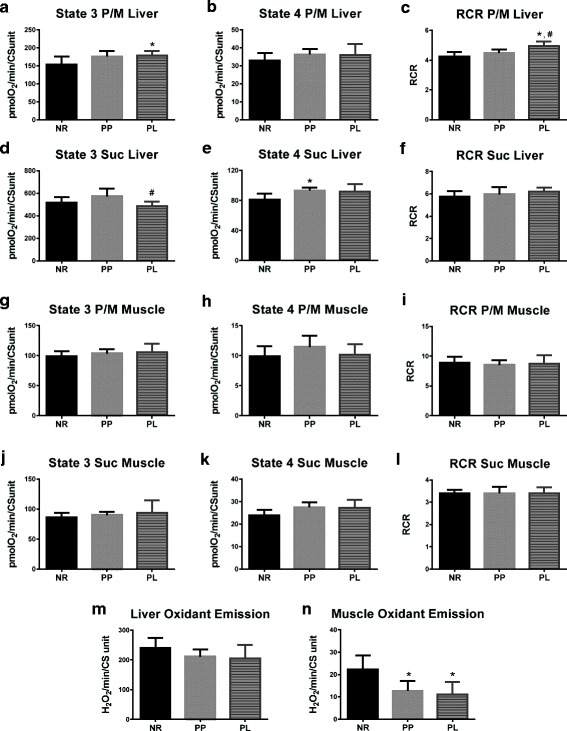
Respiration and oxidant emission from mitochondria isolated from the liver and skeletal muscle of age-matched rats that did not reproduce (NR), and rats that did not (PP) or did (PL) suckle their young for 21 days postpartum. Data include liver (**a**) state 3 respiration utilizing complex I substrates (pyruvate and malate; P/M), (**b**) state 4 respiration utilizing P/M, (**c**) respiratory control ratio (RCR) utilizing P/M), (**d**) state 3 respiration utilizing complex II substrates (succinate; suc), (**e**) state 4 respiration utilizing Suc, **f** RCR utilizing suc. Data also include skeletal muscle (**g**) state 3 respiration utilizing P/M, (**h**) state 4 respiration utilizing P/M, (**i**) RCR utilizing P/M, (**j**) state 3 respiration utilizing suc, (**k**) state 4 respiration utilizing suc, (**l**) RCR utilizing suc. Finally (**m**) oxidant emission from liver and (**n**) oxidant emission from skeletal muscle are also presented. Oxygen consumption and hydrogen peroxide (H_2_O_2_) rates were normalized to citrate synthase (CS). Data shown are mean ± SD. * indicates different from NR (*p* < 0.05), and # indicates different from PP (*p* < 0.05)

RCR is a useful tool in assessing mitochondrial function, and can be used to measure respiration capacity of the mitochondria with different available substrates. Measuring RCR using isolated mitochondria provides the ability to observe ATP phosphorylation via electron shuttling at complex I and complex II. High-resolution respirometry was used in isolated mitochondria from liver and skeletal muscle. The RCR of liver mitochondria was higher in PL compared to PP and NR rats (*p* = 0.029 and *p* = 0.002, respectively) when using pyruvate and malate as the substrate (Fig. 2c). No statistical differences were detected in liver RCR between groups when succinate was used as the substrate (*p* = 0.234; Fig. 2f). In addition, no statistical differences were detected in skeletal muscle RCR between groups when pyruvate and malate or succinate was used as the substrate (*p* = 0.792 and *p* = 0.996, respectively) (Fig. 2i and l). Oxidant emission occurs during oxidative phosphorylation in the mitochondria, primarily at complex I and complex III sites [27]. No statistically differences (*p* = 0.191) were detected in oxidant emission from mitochondria isolated from liver (Fig. 2m). Oxidant emission from mitochondria isolated from skeletal muscle was lower in PP and PL compared to NR (*p* = 0.017 and *p* = 0.008, respectively) (Fig. 2n).

### Mitochondrial complex activity

**Fig. 3:**
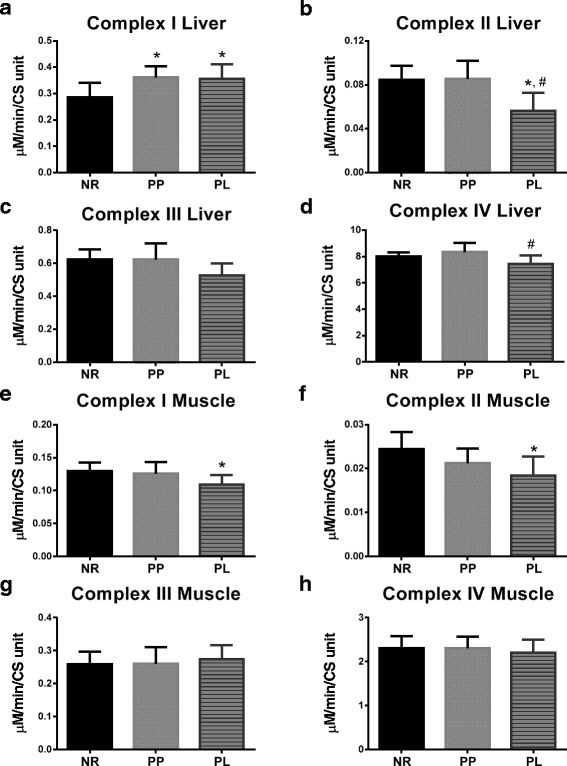
Enzymatic activity of the mitochondrial complexes for liver and skeletal muscle of age-matched rats that did not reproduce (NR), and rats that did not (PP) or did (PL) suckle their young for 21 days postpartum. Data include (**a**) complex I, (**b**) complex II, (**c**) complex III, and (**d**) complex IV activity in the liver. In addition, data include (**e**) complex I, (**f**) complex II, (**g**) complex III, and (**h**) complex IV activity in skeletal muscle. Complex activity data are normalized to citrate synthase (CS). Data shown are mean ± SD. * indicates different from NR (*p* < 0.05), and # indicates different from PP (*p* < 0.05)

Phosphorylating ADP in the mitochondria is accomplished through electron pumps that harness the energy of electrons by oxidizing the reduced form of either NADH or FADH. Specifically, complex I transfers electrons from NADH to coenzyme Q10. Complex II transfers electrons from FADH. Complex III passes electrons down the chain by reducing cytochrome c. And, complex IV converts molecular oxygen into water and further shuttles protons into the intermembrane space. Activity of complex I in liver mitochondria was higher for both PP and PL rats compared to NR rats (*p* = 0.027 and *p* = 0.046, respectively) (Fig. 3a). Conversely, enzymatic activity of complex II in liver mitochondria was lower in PL rats than both NR and PP rats (*p* = 0.004 and *p* = 0.0029, respectively) (Fig. 3b). Complex III activity in liver mitochondria of PL rats was lower, albeit not statistically different, than PP and NR rats (*p* = 0.065 and *p* = 0.075, respectively) (Fig. 3b). Complex IV activity was lower in liver mitochondria of PL compared to PP rats (*p* = 0.025; Fig. 3d). Enzymatic activity of complex I and II were lower in mitochondria isolated from skeletal muscle in PL compared to NR rats (*p* = 0.044 and *p* = 0.032, respectively). (Fig. 3e and f).

### Markers of oxidative and lipid metabolism

**Fig. 4:**
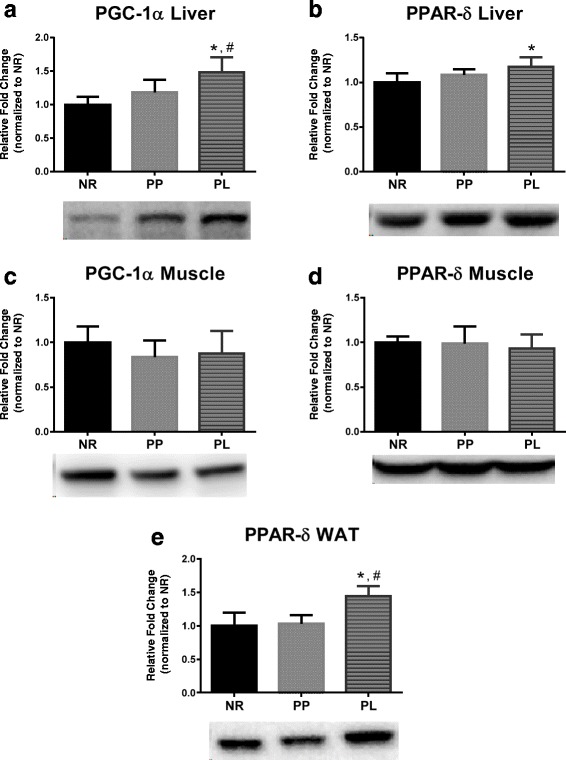
Markers of metabolism in liver, skeletal muscle, and white adipose tissue (WAT) of age-matched rats that did not reproduce (NR), and rats that did not (PP) or did (PL) suckle their young for 21 days postpartum. Data include (**a**) PGC-1α and (**b**) PPARδ protein levels in liver. (**c**) PGC-1α and (**d**) PPARδ protein levels in skeletal muscle. **e** PPARδ protein levels in WAT. Representative blots are shown under the graphs. Data shown are mean ± SD. * indicates different from NR (*p* < 0.05), and # indicates different from PP (*p* < 0.05)

The PPAR superfamily is associated with the regulation of genes involved in oxidative metabolism. Specifically PGC-1α is associated with regulation of genes involved in mitochondrial biogenesis [28] and PPARδ is associated with genes involved in lipid and glucose metabolism [29, 30]. PGC-1α liver protein expression was higher in PL rats compared to PP and NR rats (*p* = 0.035 and *p* = 0.001, respectively) (Fig. 4a). Also, PPARδ liver protein expression was higher in PL compared to NR rats (*p* = 0.009) (Fig. 4b). No statistically differences were detected for PGC-1α and PPARδ in skeletal muscle (*p* = 0.331 and *p* = 0.691, respectively) (Fig. 4c and d). PPARδ protein expression in WAT was higher in PL compared to PP and NR rats (*p* < 0.001) (Fig. 4e).

### Markers of oxidative stress

**Fig. 5:**
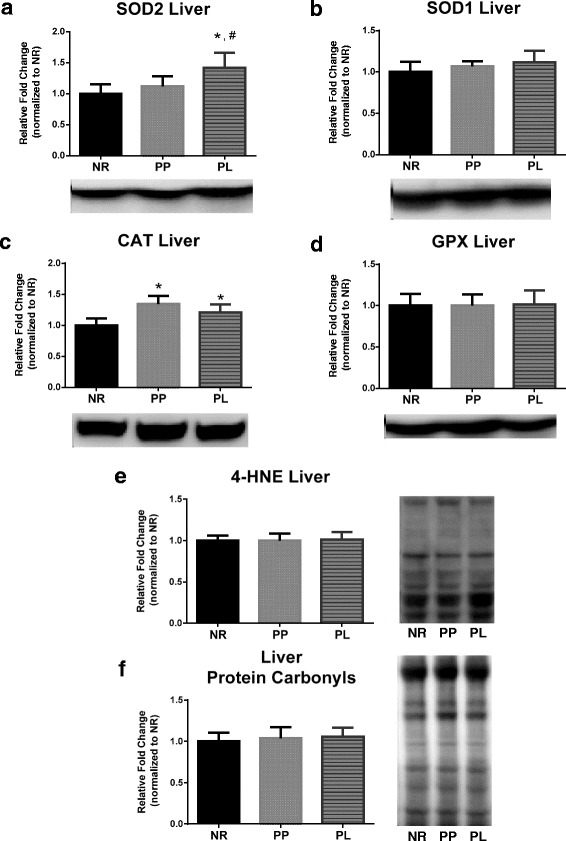
Markers of oxidative stress in liver of age-matched rats that did not reproduce (NR), and rats that did not (PP) or did (PL) suckle their young for 21 days postpartum. Data include protein level of the antioxidants (**a**) SOD2, (**b**) SOD1, (**c**) CAT, and (**d**) GPX. In addition, data include markers of oxidative damage including (**e**) lipid peroxidation determined by 4-HNE, and (**f**) protein carbonyls levels determined by using oxyblot. Representative blots are shown under the graphs (**a**-**d**) or to the right of the graphs (**e** and **f**). Data shown are mean ± SD. * indicates different from NR (*p* < 0.05), and # indicates different from PP (*p* < 0.05)

**Fig. 6:**
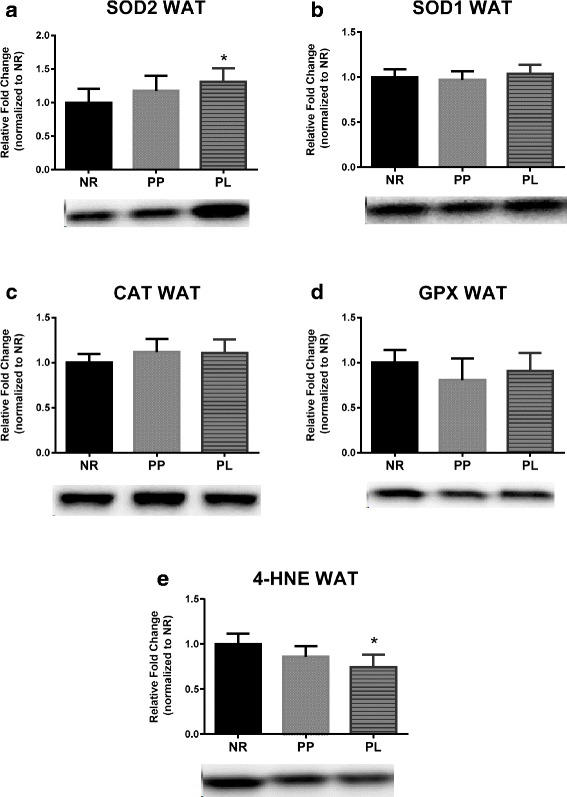
Markers of oxidative stress in white adipose tissue (WAT) of age-matched rats that did not reproduce (NR), and rats that did not (PP) or did (PL) suckle their young for 21 days postpartum. Data include protein level of the antioxidants (**a**) SOD2, (**b**) SOD1, (**c**) CAT, and (**d**) GPX. In addition, data include (**e**) lipid peroxidation determined by 4-HNE, Representative blots are shown under the graphs. Data shown are mean ± SD. * indicates different from NR (*p* < 0.05)

**Fig. 7:**
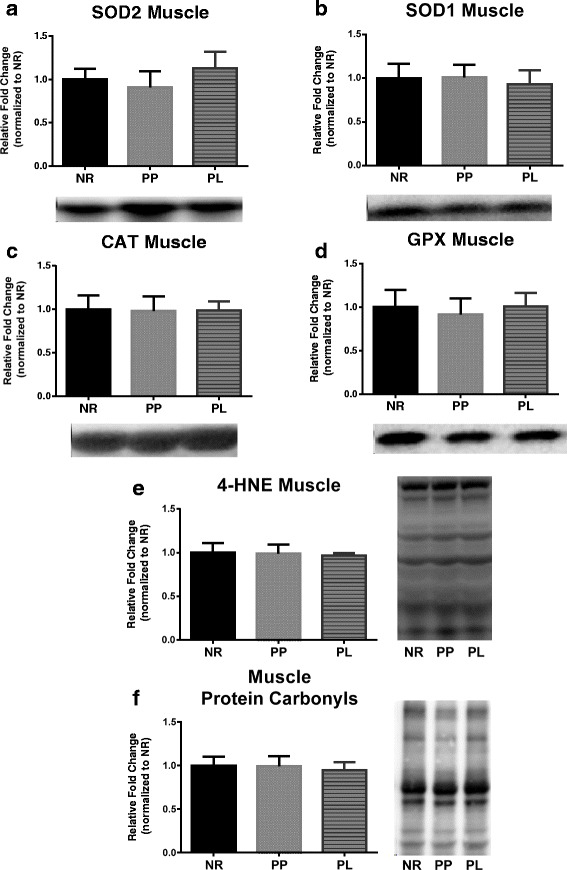
Markers of oxidative stress in muscle of age-matched rats that did not reproduce (NR), and rats that did not (PP) or did (PL) suckle their young for 21 days postpartum. Data include protein level of the antioxidants (**a**) SOD2, (**B**) SOD1, (**c**) CAT, and (**d**) GPX. In addition, data include markers of oxidative damage including (**e**) lipid peroxidation determined by 4-HNE, and (**f**) protein carbonyls levels determined by using oxyblot. Representative blots are shown under the graphs (**a**-**d**) or to the right of the graphs (**e** and **f**). Data shown are mean ± SD

The balance between oxidants and antioxidants is often referred to as oxidative stress. Endogenous antioxidant defense mechanisms exist to protect and detoxify oxidants. Specifically, SOD2 and SOD1 act to detoxify superoxide into the lesser harmful hydrogen peroxide. Hydrogen peroxide can be then be further detoxified by CAT and GPX. The protein levels of the antioxidants (SOD2, SOD1, CAT, and GPX) and markers of oxidative damage were compared between groups. Liver SOD2 protein levels were higher in PL compared to NR and PP rats (*p* = 0.001 and *p* = 0.025, respectively) (Fig. 5a). Liver CAT protein expression was higher in PL and PP rats compared to NR rats (*p* = 0.015 and *p* = 0.001, respectively) (Fig. 5c). No statistical differences (*p* > 0.05) were detected for SOD1, GPX, 4-HNE or protein carbonyls in liver (Fig. 5b and d-f). SOD2 protein levels were higher in WAT of PL compared to NR rats (*p* = 0.039) (Fig. 6a), and 4-HNE was lower in WAT of PL rats compared to NR rats (*p* = 0.004) (Fig. 6e). No statistical differences (p > 0.05) were detected for SOD2, SOD1, CAT, GPX, 4-HNE, or protein carbonyls in skeletal muscle (Fig. 7).

## Discussion

The metabolic changes that a female experiences during lactation are often the most dramatic physiological changes that she will experience in her lifetime. In this regard, many metabolic challenges (e.g., exercise, calorie restriction, etc.) have demonstrated to provide protective health benefits against diseases such as obesity and type II diabetes [1, 2, 31, 32]. Epidemiological data suggests that lactation contributes to a lower prevalence of obesity and type II diabetes, in addition to other health disparities [5–9]. Yet despite this knowledge, our understanding of the metabolic differences between females that maintain lactation and those that do not maintain lactation is limited. Our data suggest that metabolism and the bioenergetic capacity of females is enhanced following lactation in the rat. Specifically, we observed a reduced body and WAT mass and lower serum glucose concentration in animals that lactated. Furthermore, our data indicate that glucose metabolism was improved, in part, due to improved liver mitochondria function.

### Change in body and tissue mass

The process of milk synthesis presents a high energetic demand to the mother. In mice, resting metabolic rate during peak lactation has been recorded as being 2-fold higher than controls [33]. A large contribution to this increase in resting metabolic rate is imposed by the liver, which doubles in size, and the mammary gland that is actively synthesizing milk. Our findings show that PP rats had a higher body mass 28 days post-partum compared to NR, while no statistical differences were detected between PL and NR rats. Furthermore, our results indicate that while body mass was not different between NR and PL rats, WAT mass was lower in PL rats compared to both PP and NR rats. These finding agree with previous work showing lactation leads to a reduction in absolute fat mass associated with fewer visceral adipocytes [34–36]. In addition, liver is hypertrophied during gestation and lactation in order to meet the demands of higher energy utilization. Our data support this, and liver mass was still higher in PL rats 7 days after weaning, which may have masked some of the difference in body mass between the groups. These findings highlight the extent of anatomical changes that occur in response to the metabolic perturbations imposed by lactation.

### Lactation and metabolism

Systemic changes in numerous metabolic processes must occur during pregnancy for the female’s body to support the growing fetus. One such change is an increase in maternal insulin production. An increase in maternal insulin production facilitates glucose transport to the fetus, but this can often result in increased insulin resistance in maternal cells. In some cases, this may manifest itself as gestational diabetes. We show that alterations of glucose metabolism due to pregnancy is sustained to some degree in females that do not participate in lactation. This idea is supported through epidemiological findings suggesting that this effect increases a woman’s risk of developing type II diabetes later in life relative to women who do breastfeed [5, 37]. In contrast to pregnancy, there is a decrease in insulin secretion during lactation returning to pre-pregnancy levels that is associated with a decline in β-cell proliferation and improved insulin sensitivity [13].

Importantly, our finding of higher liver protein levels of PPARδ in PL rats may help explain how glucose metabolism may be increased due to a lactation period. PPARδ plays a significant role in the regulation of glucose metabolism and insulin sensitivity [29] and Sanderson and collaborators demonstrated its importance in the liver. Specifically, knockout models of PPARδ had lower expression of genes relating to glucose metabolism and higher plasma glucose in a fasted state compared to wild type mice [38]. These studies and our data, in which fasted serum glucose was decreased and liver PPARδ protein expression was higher in PL rats, suggest that the resulting modulation of PPARδ expression persists after lactation and is at least one mechanism that may confer a phenotype that protects against type II diabetes. In addition, we report that mitochondrial respiratory function was enhanced in PL rats when using complex I substrates, as indicated by a higher RCR. Considering that complex I respiration plays a large role in the utilization of glucose, via high NADH:FADH_2_ production, our findings provides a mechanistic outcome for the enhanced ability of cells in PL rats to metabolize glucose. Interestingly, measurements of enzymatic activity of the electron transport system show that both PP and PL rats had higher complex I activity. Our findings of increased liver complex I mitochondrial function in PL animals may be better explained by the increased protein expression of PGC-1α and PPARδ. While PPARδ knockout models have demonstrated that PPARδ is involved in the expression of genes relating to oxidative metabolism, PGC-1α is a well-known regulator of mitochondrial biogenesis and coactivator of genes involved in oxidative phosphorylation [28]. Thus, increased expression of these proteins likely results in the improved mitochondrial function observed. PL rats also expressed lower liver complex II activity compared to both PP and NR, and lower respiration when using complex II substrates. This down regulation of complex II respiration further limits use of FADH_2_ to fuel OXPHOS, and as a result, likely down regulates fat metabolism via beta oxidation, an effect that further supports the use of glucose as the primary fuel to support ATP production in PL rats. During pregnancy, the mother’s body experiences increasing visceral adiposity [12]. During lactation however, a shift in lipoprotein lipase and triacylglyceride levels facilitate the mobilization of fats to the mammary gland to be used for milk synthesis [14]. In contrast to PPARδ’s role in liver, PPARδ in WAT is involved in catabolic effects through oxidation of fatty acids [30]. Our data show that PPARδ protein expression is higher in WAT of PL rats compared to NR and PP rats. Our findings of increased PPARδ protein expression in two functionally different tissues also demonstrate that a systemic signaling event is a likely candidate for driving these changes.

### Oxidative stress in liver and WAT

The production of reactive oxygen species (ROS) is a naturally occurring phenomenon that can manifest itself through processes of energy production and metabolism; however an imbalance between ROS production and a counteracting antioxidant system can result in oxidative damage [39]. Oxidative stress has been implicated as a driving force in the development of diabetes and other health disparities [40, 41] and several investigators have theorized that oxidative stress is a physiological cost of reproduction that has the potential to impact a female’s future reproductive performance and longevity [42, 43]. Yet, the results of studies testing this hypothesis have provided equivocal results [18]. Our data show no differences in markers of liver lipid peroxidation and protein oxidation between the groups described here that were collected 1 week after lactation has ended, suggesting that there was no persistent oxidative cost of reproduction. With no difference in oxidant emission in liver, our finding of higher liver SOD2 in PL rats may be better explained by increased PGC-1α, as SOD2 is a downstream target of PGC-1α [44] than as a mechanism of preventing oxidative damage. Systemic effects of prior lactation are further demonstrated by our finding that SOD2 protein levels were also higher in the WAT of PL rats. However, oxidative damage was lower in WAT of PL as shown by decreased 4-HNE. Reduced oxidative damage in WAT following lactation may serve as a beneficial adaptation, as oxidative stress in WAT has been proposed to disrupt adipokine secretion by WAT, which may play a role in the development of metabolic syndrome [41].

## Conclusions

We report that rats who experienced lactation had reduced body mass, reduced WAT mass, and exhibited changes in mitochondrial function and select markers of metabolism and oxidative stress after lactation had ended and mammary tissue had regressed. Collectively these data advance our understanding of the physiological and metabolic changes that occur in animals that give birth and either suckle their pups or do not suckle their pups. In closing, these animal findings may aid future research efforts in identifying potential physiological mechanisms that mediate the persisting maternal metabolic health benefits accrued due to lactation in human populations. This topic remains an important clinical issue, as identifying these targets can be used to benefit mothers who are unable to breastfeed their young.

## Abbreviations

4-HNE: 4-hydroxynoneal
BSA: Bovine serum albumin
CAT: Catalase
GPX: Glutathione peroxidase
NEFA: Non-esterified fatty acids
NR: Non-reproductive rats
PBS: Phosphate-buffered saline
PGC-1α: Peroxisome proliferator activated receptor gamma coactivator 1 alpha
PGC-1β: Peroxisome proliferator activated receptor gamma coactivator 1 beta
PL: Rats that allowed to mate, became pregnant, and suckled their young until weaning at 21 days
PP: Rats that allowed to mate and became pregnant, but were not allowed to suckle their pups
PPAR-α: Peroxisome proliferator activated receptor alpha
PPARδ: Peroxisome proliferator activated receptor delta
RCR: Respiratory control ratio
SOD1: Superoxide dismutase 1
SOD2: Superoxide dismutase 2
WAT: White adipose tissue
